# Ovariotomy—Sequel of Dr. Russell’s Case

**Published:** 1870-03

**Authors:** J. F. Kelsey


ARTICLE IX.

### OVARIOTOMY—SEQUEL OF DR. RUSSELL'S CASE.

BY J. F. KELSEY, M.D.

The case to which I allude is that of a Mrs. Dearth, from whom Dr. R. removed a large ovarian tumor, in the month of December, 1868, which was duly reported in the CHICAGO MEDICAL EXAMINER, for March, 1869.

I saw her during the spring of 1869. Found her in comparative health. Examined the wound and found that it had not healed soundly up; that at two or three points where the large pedicle was embodied, there was fungoid growths, from the size of a pea to that of a good-sized bean, discharging a little saneous fluid. Ordered them to be cauterized with sulphate of copper and dressed with carbolic acid ointment, with the hopes of healing them up. During the months of May, June, and July, her health rapidly improved, gaining in strength and flesh until she resumed her household duties, and was able to walk half a mile to church, and back again, without over-fatigue. I saw her again the 1st of September, and learned that the wound had never entirely healed; that the morbid growths were increasing in size and numbers; abdomen tender; that about the 25th of August, she, while in the act of dipping water from a rain-trough, slipped, her person falling violently upon the edge of the trough; and, as she expressed herself, had not seen a well day since.

*Sep. 16th.* Called in great haste to see her, and found the following conditions present in her case. Countenance pale and anxious; lips thin and retracted; great emaciation of flesh; pulse quick and feeble; tongue clean but reder than usual; great pain in the region of the wound; bowels constipated; vomiting every few minutes a large amount of stercoraceous matters; abdomen tympanitic and tender. The wound had become a nodular mass of small tumors (15 .to 20), from the size of a pea to that of a hen's egg. Interspersed around its margin, some three or four of them were isolated, from two to four inches from the margin of the wound into the walls of the abdomen.

Of course, the prognosis was unfavorable, and the treatment could be but palliative.

This state of things kept up to a greater or less degree until she died, November 3d, 1869, 10 months and 18 days from the time of operation. That she died of more or less peritoneal inflammation, and that mechanical injury was an exciting cause of that inflammation, is quite apparent. But upon closer examination, I shall attribute its pathology from a different standpoint, which, if correct, shows too plainly how a slight mistake may turn the scale of life, and, if not revealed, its ignorance would be buried with the dead.

It will be remembered by every assistant at the operation that there were three pedicles to the tumor; that the two small ones were materially different in their texture and appearance; that their origin were separate and distinct from the large one, being lower in the pelvis; that they were very vascular, requiring a firm application of the ligature to restrain hemorrhage; while the large one partook of the character of the tumor itself, without any disposition to hemorrhage whatever; that in its separation, it had the exact appearance of the walls of the tumor itself, full of deep sinuses, etc.; that its point of attachment extended from the sacro-vertebral angle, directly upward, a distance of three or four inches—a region not likely to be the true seat of the ovaries. Now, supposing that this had been a portion of the walls of the tumor and not of itself a true pedicle, it being firmly adhered to the side of the spinal column, would it not readily maintain its vitality and engraft itself into the abdominal muscles, during the process of cicatrization? and, if so, would there not be an attempt to a reproduction of cell-growth, producing morbid changes of an amorphus character, even in the walls of the abdomen itself? Then, if we admit this to be the fact, as it appears to be in her case, we readily perceive the germs of destruction which in the end could but prove fatal, although her fall might have been an exciting cause to hasten her untimely death.

